# Generative Deep Neural Networks for Inverse Materials Design Using Backpropagation and Active Learning

**DOI:** 10.1002/advs.201902607

**Published:** 2020-01-09

**Authors:** Chun‐Teh Chen, Grace X. Gu

**Affiliations:** ^1^ Department of Materials Science and Engineering University of California Berkeley CA 94720 USA; ^2^ Department of Mechanical Engineering University of California Berkeley CA 94720 USA

**Keywords:** composites, inverse problem, machine learning, materials design, optimization algorithms

## Abstract

In recent years, machine learning (ML) techniques are seen to be promising tools to discover and design novel materials. However, the lack of robust inverse design approaches to identify promising candidate materials without exploring the entire design space causes a fundamental bottleneck. A general‐purpose inverse design approach is presented using generative inverse design networks. This ML‐based inverse design approach uses backpropagation to calculate the analytical gradients of an objective function with respect to design variables. This inverse design approach is capable of overcoming local minima traps by using backpropagation to provide rapid calculations of gradient information and running millions of optimizations with different initial values. Furthermore, an active learning strategy is adopted in the inverse design approach to improve the performance of candidate materials and reduce the amount of training data needed to do so. Compared to passive learning, the active learning strategy is capable of generating better designs and reducing the amount of training data by at least an order‐of‐magnitude in the case study on composite materials. The inverse design approach is compared with conventional gradient‐based topology optimization and gradient‐free genetic algorithms and the pros and cons of each method are discussed when applied to materials discovery and design problems.

## Graphical abstract

A general‐purpose inverse design approach using generative inverse design networks (GIDNs) is proposed. This deep neural network–based approach uses backpropagation and active learning for inverse design. It is shown that GIDNs can overcome local minima traps and be widely applied to materials discovery and design problems.

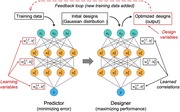

## 1 Introduction

Conventional materials discovery and design processes are mostly based on expensive, laborious, and time‐consuming trial‐and‐error approaches. The outcome of this Edisonian‐type approach not only depends strongly on human intuition and domain expertise but also a bit of luck. Consequently, the rational design of materials with superior properties is the ultimate goal of modern engineering applications. Recently, with rapid advances in artificial intelligence (AI) and computing power, machine learning (ML) techniques have improved the state‐of‐the‐art in automated image classification, natural language processing, speech recognition, and many other exciting domains.^1^ In materials science, ML techniques give computers the ability to learn structure–property relationships of materials from experimental or simulation data. With proper ML algorithms, suitable material descriptors (e.g., configuration, topology, or fingerprint), and enough training data, ML models could be trained for predicting the properties of candidate materials. This data‐driven approach has shown high potential and wide applicability to accelerate and simplify materials discovery and design processes in a manifold way. While the ML‐based methodology for materials discovery and design is still in the early stages of research, a large number of attempts were made by numerous research groups in recent years, in which a wide range of applications across length and time scales were considered, from atomistic‐scale molecular compounds discovery to macroscale composite materials design.^2^ Most ML‐based applications in materials science today, however, were dedicated to training ML models to predict the property of interest (as outputs) using material descriptors (as inputs).^3^, ^4^, ^5^, ^6^ In those applications, the emphasis was almost always on the selection of ML algorithms or material descriptors for mapping an input space to an output space. After training, ML models could be used as filters to explore a predefined design space in a brute‐force manner to search for promising candidate materials based on some design criteria.^5^, ^6^ Needless to say, using ML models to predict the properties of new materials is much more efficient than synthesizing the materials and measuring their properties in a laboratory. This ML‐based approach for rapid predictions of material properties also has computational advantages over physics‐based modeling tools, such as density functional theory (DFT), molecular dynamics (MD), or the finite element method (FEM), by which various material properties can be described by solving complex governing equations. Typically, compared to physics‐based modeling tools, predictive ML models could offer computational speedup by several orders of magnitude.^7^, ^8^, ^9^ Nevertheless, brute‐force screening can only be applied to problems with a small design space. For more complex materials discovery and design problems, the number of possible candidate materials could easily reach an astronomical number. For instance, consider a composite FEM model made up of two different base materials. This model needs no more than 300 elements (design variables) to make the number of possible combinations more than the number of atoms in our observable Universe (assumed to be around 10^80^). Other examples can also be found in protein engineering, drug discovery, and others.^10^ In a vast design space, it is infeasible to use forward modeling tools, even including predictive ML models, to explore all possible combinations—no matter how much computing power is available or how efficient the forward modeling tool is, the enormous complexity makes brute‐force screening impossible. A fundamental bottleneck is the lack of robust inverse design approaches to identify promising candidate materials without exploring the entire design space.

For design problems with large numbers of variables to be optimized, gradient‐based optimization methods (gradient descent), such as steepest descent and conjugate gradient, are generally very efficient in searching for optimal designs, which minimize an objective function subject to a set of constraints. Gradient‐based optimization methods have been widely applied to major engineering industries, in which those methods are often referred to as topology optimization. A common goal in topology optimization problems is to search for optimal material distributions to maximize the performance of structural parts such as aircraft and automotive components, buildings, and others.^11^ Despite the successes of gradient‐based optimization methods, those methods face several challenges when applied to materials discovery and design problems: (a) the local minima problem. Most optimization problems of interest in engineering and scientific fields involve many critical points including local minima, saddle points, and discontinuities. Gradient descent is known to often get stuck at critical points where the gradient has vanished. Therefore, the solutions obtained using a gradient‐based optimization method may have inferior performance due to a bad selection of initial values in the optimization, and how to select good initial values is unfortunately quite challenging and problematic; (b) the calculation of analytical gradients. For most topology optimization applications, the goal is often to search for an optimal material distribution to minimize compliance (maximize stiffness) for a given loading condition and volume fraction constraint. In those problems, the analytical gradients could be calculated using adjoint methods.^12^ Compared to numerical gradients, analytical gradients are exact and the calculation requires much less computational cost. However, the calculation of analytical gradients is nontrivial and only possible when knowing the explicit form of the optimization problem as well as the governing equations and the algorithm that is used to solve the equations. Therefore, it is impractical to calculate analytical gradients for most materials discovery and design problems. On the other hand, finite‐difference methods (FDM) are commonly used to calculate numerical gradients when the calculation of analytical gradients is infeasible. However, the calculation of numerical gradients is computationally expensive. Consequently, when the number of design variables is large, the calculation of numerical gradients is often the bottleneck in the optimization and the results are subject to inaccuracies; (c) the availability of objective functions. For materials discovery and design problems using large‐scale materials data and informatics in existing databases, such as the materials project, automatic flow for materials discovery, open quantum materials database, and novel materials discovery,^6^, ^13^ even numerical gradients cannot be calculated since the objective function in terms of design variables does not exist.

In this work, we present a general‐purpose inverse design approach using generative inverse design networks (GIDNs). Autoencoders and generative adversarial networks (GANs)^14^ are two of the most commonly used and efficient generative models. Nevertheless, those generative models by themselves face difficulties when it comes to generating designs “better” than training samples, making them not suitable for materials discovery and design problems. Autoencoders compress a high‐dimensional input (e.g., images) to a low‐dimensional vector (known as the latent space) and transform the vector back into the same dimension as the input. Thus, autoencoders are trained to reproduce the training data by learning a low‐dimensional representation of the data. Instead of learning how to compress data, GANs are trained to generate new data similar to training data. However, in materials discovery and design problems, the main purpose is to generate new candidate materials that have better performance than the training samples. Both generative models were not proposed for this purpose. The proposed inverse design approach uses deep neural networks (DNNs) to construct an objective function in terms of design variables. This inverse design approach can be applied to any materials discovery and design problem. After training, the analytical gradients of an objective function with respect to design variables are calculated using backpropagation. Unlike the computation of numerical gradients using FDM, backpropagation uses the chain rule to calculate analytical gradients. In materials discovery and design problems, the numerical gradient with respect to each design variable has to be calculated individually when using FDM. On the other hand, backpropagation can calculate all the analytical gradients simultaneously by using a forward and backward pass through the neural network. It is of note that using backpropagation to calculate gradients with respect to input features in DNNs is not a new technique. Sensitivity analysis using the same technique has been regularly applied to identify the most important input features in many ML‐based applications.^15^ For instance, a sensitivity analysis was applied to explain the diagnostic prediction of cancers^16^ and the classification of images by DNNs.^17^ Recently, the same technique was also applied to generate adversarial examples.^18^ However, to the best of our knowledge, very few attempts were made to design materials using backpropagation. While other works in the literature used generative models for inverse design,^19^ the novelty of the proposed inverse design approach includes: (a) it uses random initialization of design variables based on a Gaussian distribution to overcome the local minima problem. For design problems with large numbers of variables, the number of possible combinations could be near‐infinity but the number of local minima in the design space is much less.^20^ If we can capture as many local minima as possible, the chance to find global minima will be higher; (b) it uses active learning to improve the performance of promising candidate materials and reduce the amount of training data needed to do so. Deep learning approaches are data‐hungry. However, preparing a large amount of training data could be challenging in many cases. Thus, to obtain better designs using less amount of training data is crucial to accelerate materials discovery and design processes. A composite design problem is chosen as a case study to demonstrate the proposed inverse design approach. We compare it with other common optimization methods including conventional gradient‐based topology optimization and gradient‐free evolutionary (genetic) algorithms and show advantages of the proposed inverse design approach over the other optimization methods.

## 2 Results and Discussion

### 2.1 Generative Inverse Design Networks

**Figure 1:**
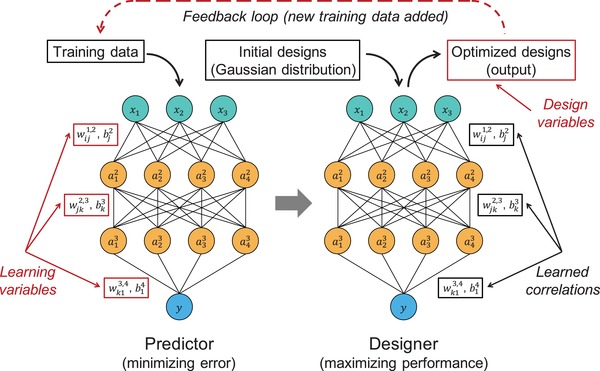
The framework of Generative Inverse Design Networks (GIDNs). GIDNs consist of two DNNs: the predictor and the designer. In the predictor, the weights and biases are learning variables. Those values are optimized to minimize the difference between the ML predictions and ground truth. In the designer, the values of the weights and biases are adopted from the predictor and set to be constants. Initial designs generated with values from a Gaussian distribution are fed into the designer as inputs and optimized designs are generated as outputs. In the feedback loop, the optimized designs are verified by a physics‐based model and can be added to previous training data for the next iteration of training and design processes.

The framework of our inverse design approach using GIDNs is depicted in **Figure** **1**. GIDNs consist of two DNNs: a “predictor” and a “designer.” Both DNNs have the same neural network structure. The hyperparameters including the number of hidden layers and neurons are tuned to balance the prediction accuracy and computational cost (see the Supporting Information). The predictor is a forward predictive model trained to approximate a physics‐based model (or an arbitrary function). The learning variables in the predictor are the weights and biases connecting the neurons in adjacent layers. After training, the values of the weights and biases in the predictor are assigned to the designer. Unlike the predictor, the designer is an inverse design model, in which the weights and biases are no longer learning variables but constants. The learning variables in the designer are set to be the design variables. Thus, the training process for the designer becomes a design process to maximize (or minimize) the desired property (or properties). Initial designs with values from a Gaussian distribution are fed into the designer as inputs. The optimized designs are then generated as outputs based on analytical gradients calculated using backpropagation. In the feedback loop, the optimized designs are verified by a physics‐based model and can be added to previous training data for the next iteration of training and design processes.

Before applying GIDNs to highly complex design problems, we choose a simple function of two variables (so‐called the peaks function) as an example to demonstrate the proposed inverse design approach. The formula of the peaks function is

(1) $$z=3{\left(1-x\right)}^{2}e^{\left(-x^{2}-{\left(y+1\right)}^{2}\right)}-10\left(\frac{x}{5}-x^{3}-y^{5}\right)e^{\left(-x^{2}-y^{2}\right)}-\frac{1}{3}e^{\left(-{\left(x+1\right)}^{2}-y^{2}\right)}$$

**Figure 2:**
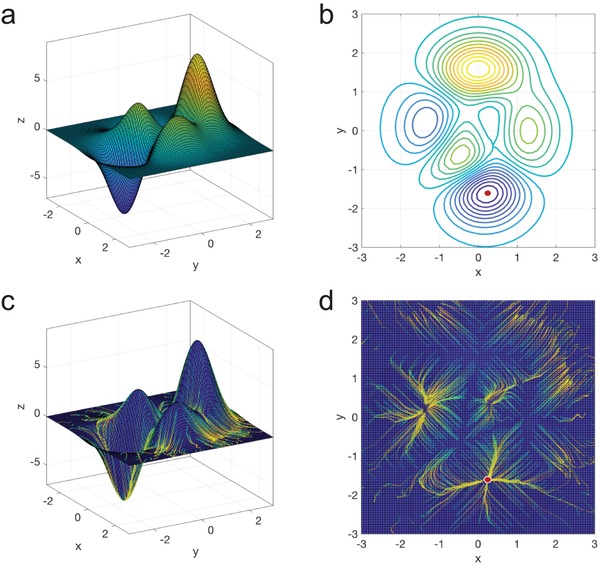
The nonconvex surface of the peaks function and the solution paths obtained using GIDNs. a) The 3D landscape of the peaks function shows several hills and valleys; b) The contour plot of the peaks function shows several local minima and maxima in the functional space. The red dot represents the location of the global minimum; c) The 3D solution paths obtained by using GIDNs starting with 1000 random initial points. The beginning of the solution paths is shown in green and the end is shown in yellow; d) The solution paths shown in 2D. Many of the solution paths are converged to the global minimum (red dot).

To understand the functional space of the peaks function, its landscape and contour are shown in **Figure** **2**a,b, respectively. As can be seen in the figures, the peaks function has a global minimum (near *x* = 0.23, *y* = −1.63) and several critical points. To search for the global minimum without exploring the entire functional space, a common approach is to use gradient descent, in which gradients can be calculated numerically using FDM. However, gradient descent is known to be limited by the local minima problem. Therefore, the optimization solution would vary with the selection of the initial point where the optimization starts with. For instance, when choosing the origin point (*x* = 0, *y* = 0) as the initial point, the optimization solution using gradient descent is most likely to get stuck at a nearest critical point (*x* = 0.29, *y* = 0.32) as shown in Figure S1 in the Supporting Information. Here, we apply GIDNs to search for the global minimum of the peaks function. The predictor is first trained to approximate the peaks function by learning the correlation between the input variables (*x* and *y*) and output variable (*z*) in Equation (1). After training, the designer is applied to search for the global minimum by using backpropagation starting with 1000 random initial points (see the Supporting Information). The 3D and 2D visualizations of the solution paths are shown in Figure 2c,d, respectively. As backpropagation used in the designer is a gradient‐based technique, optimization solutions may still get stuck at local minima. However, backpropagation calculates analytical gradients instead of numerical gradients and thus it is much faster than other numerical techniques such as FDM. Consequently, compared to other numerical techniques, GIDNs can perform much more optimizations with the same computational resource. This computational advantage of calculating gradients will be more significant when applying GIDNs to highly complex design problems, in which numerical gradients are computationally expensive to calculate. After running 1000 inverse designs with different initial values, many of the solution paths are converged to the global minimum (Figure 2c,d), showing that using random initialization of design variables is effective for solving the local minima problem.

### 2.2 Inverse Design for Composite Materials

After the success of finding the peaks function's global minimum using GIDNs, we aim to evaluate the performance of the proposed inverse design approach on highly complex materials discovery and design problems. An ideal material system for this investigation needs to have a vast design space as well as no human and measurement errors in training data to avoid ML algorithms being misled. The case study that we choose to investigate is a composite system made up of two base materials (stiff and soft) with a volume fraction constraint under Mode I fracture. Mode I is a normal‐opening mode, in which the tensile stress at the crack tip is normal to the plane of the crack and tends to open up the crack. It is the most common fracture mode and also the most dangerous one. However, it is found that the stress and strain concentration at the crack tip could be largely reduced by arranging stiff and soft materials into a specific topology.^4^, ^8^, ^9^ Thus, the toughness of the composite can be significantly increased, making it less vulnerable to Mode I fracture. The goal of this composite design problem is to search for the optimal design to maximize toughness for a given volume fraction of stiff and soft materials. In this composite design problem, the geometrical variations of composites can be systematically generated and their mechanical properties can be evaluated using FEM (see Experimental Section and Supporting Information). Therefore, in addition to any physical interpretation or practical application that this case study may provide, it can be considered as a general case of nonlinear, nonconvex, constrained materials discovery, and design problems.

**Figure 3:**
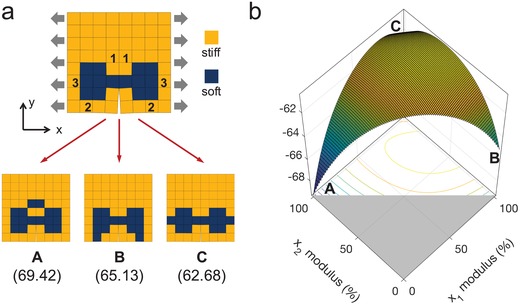
A composite design problem with a volume fraction constraint under Mode I fracture. a) An 8 by 8 composite system made up of stiff and soft base materials. Stiff and soft materials are shown in yellow and blue, respectively. The composite on the top is the optimal design with a volume fraction of 21.875% for high toughness. The composites on the bottom are three possible designs with a volume fraction of 25%, denoted by Composite‐A, Composite‐B, and Composite‐C. The numbers below indicate the corresponding toughness values; b) The optimization surface and contour plot of the composite design problem with a volume fraction constraint of 25%. The horizontal axes represent the modulus ratios of Element‐1 and Element‐2 and are denoted by *x*_1_ and *x*_2_, respectively. The modulus ratio of Element‐3, denoted by *x*_3_, is a dependent variable as the summation of *x*_1_, *x*_2_, and *x*_3_ is 200% due to the volume fraction constraint. The vertical axis represents the objective value, which is set to be the negative of the toughness. Composite‐A, Composite‐B, and Composite‐C represent the three boundary points in the optimization surface.

To understand the complexity of this composite design problem, a small composite system with 8 by 8 elements is investigated. Each element can be either stiff or soft materials. Therefore, the number of possible combinations in this 8 by 8 composite system is 2^32^ (4294967296) when considering geometrical symmetry. The optimal designs with different volume fractions for high toughness were identified using a brute‐force search in our previous work.^8^ Here, the optimal design with a volume fraction of 21.875% is shown in **Figure** **3**a. The volume fraction is denoted as the number of soft elements divided by the total number of elements in a composite. Assuming that we decide to generate the optimal design with a volume fraction of 25% by adding more soft elements (at symmetric locations). This appears like a simple task but it is nontrivial when using gradient‐based optimization methods. To be able to visualize the optimization surface, we only consider three possible stiff elements to be replaced with soft elements. Those stiff elements are at three spatial locations and denoted by Element‐1, Element‐2, and Element‐3, written on the element itself (Figure 3a). Accordingly, three possible designs with a volume fraction of 25% can be generated, which are denoted by Composite‐A, Composite‐B, and Composite‐C, and their toughness values are shown in Figure 3a. Note that the toughness values reported in this work are normalized by the toughness of a composite made up of all stiff (or soft) material. Thus, the toughness values are unit‐less. To be able to calculate gradients, we allow the modulus of the elements to vary continuously from 100% (as the stiff material) to 0% (as the soft material). The optimization surface of this composite design problem with a volume fraction constraint of 25% is shown in Figure 3b. The objective value is set to be the negative of the toughness. Therefore, searching for the composite design with the maximum toughness value is equivalent to searching for the composite design with the minimum objective value in the optimization surface. It can be seen in the figure that, depending on the initial values adopted in gradient descent, the optimization solution could be converged to any of those three possible designs. Consequently, in this composite design problem, using gradient descent cannot guarantee to find the optimal solution since the problem is nonconvex. Note that we use a small composite system and only consider three design variables to demonstrate the nonconvex nature of this composite design problem; its complexity will only increase with the number of design variables.

**Figure 4:**
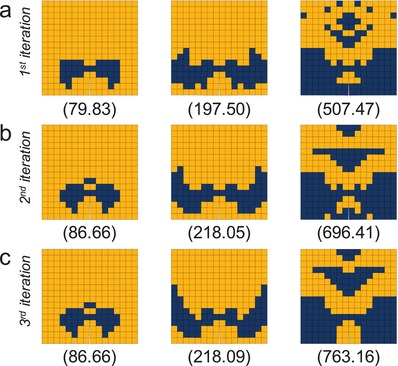
The inverse designs generated using GIDNs for a 16 by 16 composite system. a) The inverse designs generated in the first‐iteration design process for the volume fractions of 12.5%, 25%, and 50% (left to right), respectively. The numbers below indicate the corresponding toughness values; b) The inverse designs generated in the second‐iteration design process; c) The inverse designs generated in the third‐iteration design process.

Here, we apply GIDNs to this composite design problem. To increase the complexity, a larger composite system with 16 by 16 elements is investigated. This 16 by 16 composite system has a vast design space with a total of 2^128^ (about 3.4 × 10^38^) possible combinations, in which the optimal designs are impossible to identify using a brute‐force search. Due to the nonconvex nature as discussed above, it is also challenging to search for the optimal designs using conventional optimization methods. Three different volume fractions are considered: 12.5%, 25%, and 50%. In the training process, 1 000 000 composite designs for each volume fraction are randomly generated and their toughness values are calculated using FEM. 800 000 of them are used as training samples to train the predictor, and the rest 200 000 are used as testing samples to evaluate its accuracy. After training, the values of the learning parameters in the predictor are assigned to the designer. In the design process, 1 000 000 initial designs are generated from a Gaussian distribution, in which the mean value is set to match a predetermined volume fraction and the standard deviation is set to be 0.25. The initial designs are then fed into the designer as inputs. The outputs of the designer are optimized designs, in which the design variables are optimized based on analytical gradients calculated using backpropagation. The optimized designs with the highest toughness (calculated using FEM) for different volume fractions are shown in **Figure** **4**a.

The optimized designs in Figure 4a, however, cannot be guaranteed to be the best designs (global minima). A strategy to increase the performance of outputs generated by the designer is to train the predictor with more training samples to increase the prediction accuracy. We find that when increasing the number of training samples from previously 800 000 to 24 000 000, the top performance increases by 1.3%, 5.4%, and 23.4%, for the volume fractions of 12.5%, 25%, and 50%, respectively (Figure S2, Supporting Information). The relationship between the number of training samples and the performance of inverse designs for the 8 by 8 composite system is discussed in the Supporting Information. Nevertheless, preparing more training samples is time‐consuming and impractical in many applications. Since the goal of this composite design problem is to search for high‐performing designs, the prediction accuracy for low‐performing designs is less important. For this reason, we use an active learning strategy by adding high‐performing samples with their toughness values verified by FEM to previous training samples for the next iteration of training and design processes. Specifically, 800 000 of the optimized designs generated by the designer are added to previous training samples and the rest 200 000 are added to previous testing samples. Thus, in the second‐iteration training process, a total of 1 600 000 training samples are used to train the predictor and 400 000 testing samples are used to evaluate its accuracy. In the second‐iteration design process, 1 000 000 initial designs generated from a Gaussian distribution are fed into the designer as inputs to generate new optimized designs as outputs. The new optimized designs with the highest toughness for different volume fractions in the second‐iteration are shown in Figure 4b.

Compared to the first‐iteration designs (Figure 4a), the top performance of the second‐iteration designs (Figure 4b) increases by 8.5%, 10.4%, and 37.2%, for the volume fractions of 12.5%, 25%, and 50%, respectively. The result confirms our hypothesis that training the predictor with more high‐performing samples can increase the probability for the designer to identify better designs. Thus, we repeat this active learning strategy in the third‐iteration training and design processes. The new optimized designs with the highest toughness for different volume fractions in the third‐iteration are shown in Figure 4c. Compared to the second‐iteration designs (Figure 4b), the top performance of the third‐iteration designs (Figure 4c) does not increase much. For the volume fraction of 12.5%, the best designs in the second‐interaction and third‐interaction are the same. For the volume fractions of 25% and 50%, the top performance of the third‐iteration designs only increases by 0.02% and 9.6%, respectively. Note that the third‐iteration designs (Figure 4c) are generated by using the active learning strategy to train with 2 400 000 samples, in which 800 000 of them are randomly generated and the rest 1 600 000 are high‐performing designs generated by the designer. It is shown that the optimized designs generated by using active learning (Figure 4c) have higher performance than those generated by using passive learning to train with 24 000 000 randomly generated samples (Figure S2, Supporting Information). Compared to passive learning, this active learning strategy uses an order of magnitude fewer training samples and generates even better designs.

As a baseline, we compare GIDNs against other optimization methods. Here, a gradient‐based topology optimization method is applied to the same composite design problem. The objective function, which is set to be the negative of the toughness, is evaluated using FEM and the gradients of the objective function with respect to the design variables are calculated numerically using FDM with the central difference approximation (see the Supporting Information). The optimized designs generated using a gradient‐based topology optimization method are shown in Figure S3 in the Supporting Information. The performance of those optimized designs is approximately 21.3%, 27.7%, and 58.0% lower than that of the optimized designs generated using GIDNs (Figure 4c) for the volume fractions of 12.5%, 25%, and 50%, respectively. Furthermore, a binary genetic algorithm is applied to the same composite design problem. Genetic algorithms were inspired by the natural evolution process and commonly used to produce multiple solutions for optimization and design problems. The fitness function, which is set to be the toughness, is evaluated using FEM (see the Supporting Information). As there is no standard technique to consider a volume fraction constraint for binary genetic algorithms, the volume fraction constraint is removed. The optimizations using a binary genetic algorithm are converged after 1000 successive generations (Figure S4, Supporting Information) and the optimized designs are shown in Figure S5 in the Supporting Information. Although no volume fraction constraint is applied in the optimizations (larger design space), the top performance of those optimized designs is lower than that of the optimized designs generated using GIDNs (Figure 4c). Lastly, we compare GIDNs against the ML‐based design approach using logistic regression in our previous work. The optimized designs for the volume fraction of 12.5% reported in our previous work^4^ are shown in Figure S6 in the Supporting Information. The top performance of those optimized designs is 8.1% lower than that of the optimized design generated using GIDNs for the same volume fraction (Figure 4c).

It is of note that each optimization method has its strengths and limitations. Gradient‐based optimization methods are efficient for problems with large numbers of design variables but can easily get stuck at local minima. Genetic algorithms are more suitable for discrete design variables but are inefficient for problems with large numbers of design variables. Those methods, however, require constantly running physics‐based simulations (FEM in this case study) to evaluate the objective function or fitness function in each optimization step. GIDNs, on the other hand, do not require running physics‐based simulations during the design process. After training, GIDNs can generate optimized designs directly based on the discovered hidden patterns and correlations learned from training data. Although it is recommended to verify the performance of optimized designs after the design process to identify the best designs, verification is not required during the design process. This advantage would be beneficial to researchers working on data‐intensive materials research where they mostly do not have the facilities to conduct massive materials simulations or experiments but have access to large‐scale materials data and informatics in existing databases. With GIDNs, the researchers can propose promising candidate materials based on large‐scale materials data and informatics without conducting any new simulation or experiment. The composite design problem demonstrated here is focused on mechanical properties; however, GIDNs can be applied to a wide range of other physical disciplines, including fluids, acoustics, thermal, and many others.

### 2.3 Statistical Analysis of ML Results

**Figure 5:**
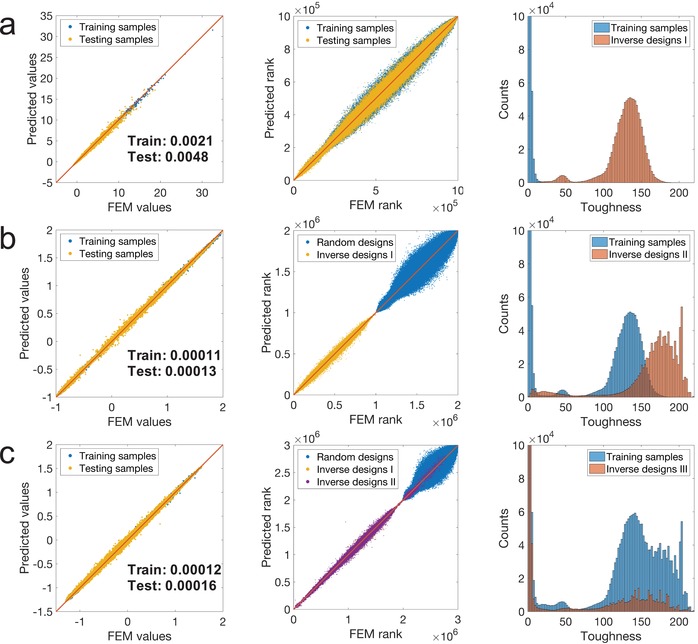
The statistical analysis results of prediction accuracy and inverse design performance. a) The statistical analysis results for the first‐iteration training and design processes. The subfigures from left to right are the comparison of the ML predicted values and FEM values, the comparison of the ML predicted ranking and FEM ranking, and the histogram of the training samples and inverse designs. b) The statistical analysis results for the second‐iteration training and design processes. c) The statistical analysis results for the third‐iteration training and design processes.

A statistical analysis is performed to evaluate the performance of GIDNs. The results for the volume fraction of 25% in the first‐iteration training and design processes are shown in **Figure** **5**a. As can be seen in the figure, the training and testing errors are both very low (0.0021 and 0.0048) and the ML predicted ranking is close to FEM ranking. The results indicate that the predictor has a high prediction accuracy and is not over‐fitted. The training sample distribution shows that the majority of the randomly generated samples used to train the predictor have very low toughness. The maximum and mean toughness values of the training samples are 61.01 and 1.86, respectively. However, the first‐iteration inverse designs show orders‐of‐magnitude improvement. The maximum and mean toughness values of the inverse designs are 197.50 and 129.54, respectively. Note that the histograms of the training samples and inverse designs are truncated for clarity and the full histograms are shown in Figure S7 in the Supporting Information. Furthermore, the statistical analysis results in the second‐iteration training and design processes are shown in Figure 5b. As with the first‐iteration results (Figure 5a), the training and testing errors are both very low (0.00011 and 0.00013). In the comparison of the ML predicted ranking and FEM ranking, it can be seen that the randomly generated samples and the first‐iteration inverse designs form two clusters, revealing that those two groups of training samples share no overlap. As the training samples in the second‐iteration contain the first‐iteration inverse designs, the maximum and mean toughness values of the new training samples increase to 197.50 and 65.70, respectively. With more high‐performing training samples, the designer can generate better designs with higher performance. The maximum and mean toughness values of the second‐iteration inverse designs increase to 218.05 and 163.59, respectively.

Lastly, the statistical analysis results in the third‐iteration training and design processes are shown in Figure 5c. As with the previous results (Figure 5a,b), the training and testing errors are both very low (0.00012 and 0.00016). However, it can be seen in the comparison of the ML predicted ranking and FEM ranking, the second‐iteration inverse designs are partially overlapped with the first‐iteration inverse designs, showing that many of the second‐iteration inverse designs are not new designs but the same as the first‐iteration inverse designs. The reason is that the number of local minima is not that many in the design space. Thus, the same optimized designs could be generated multiple times even starting with different initial designs. As the training samples in the third‐iteration contain the first‐iteration and second‐iteration inverse designs, the maximum and mean toughness values of the new training samples increase to 218.05 and 98.33, respectively. Although the maximum toughness value of the third‐iteration inverse designs increases to 218.09, the mean toughness value decreases to 51.27. The decrease in the mean toughness value is due to the bias distribution of the training samples. The training samples in the third‐iteration contain mostly high‐performing samples. Thus, the models' prediction accuracy in the high‐performing design space is improved. However, the prediction accuracy in the low‐performing design space is sacrificed. As the initial designs adopted in the design process are randomly generated, they are mostly low‐performing designs. The low prediction accuracy in the early stage of the design process misleads the optimization paths and causes the low‐performing outputs. Thus, how to improve the models' prediction accuracy in the high‐performing design space without sacrificing the prediction accuracy in the low‐performing design space is an important task for future studies. The statistical analysis results for the lower (12.5%) and higher (50%) volume fractions are shown in Figures S8 and S9 in the Supporting Information, respectively.

## 3 Conclusions

We present a general‐purpose inverse design approach using GIDNs. This inverse design approach uses backpropagation to calculate the analytical gradients of an objective function with respect to design variables. Compared to other numerical techniques such as FDM, backpropagation is much faster and accurate. Furthermore, this inverse design approach is integrated with random initialization of design variables to overcome the local minima problem and paired with the active learning strategy to improve the performance of optimized designs and reduce the amount of training data needed to do so. We apply GIDNs to design superior composite materials with high toughness and show that the performance of the inverse designs can be improved using the active learning strategy. Compared to passive learning, this active learning strategy can generate better designs with training data at least one order of magnitude less. We compare GIDNs with gradient‐based topology optimization and genetic algorithms and show that GIDNs outperform those methods in our case study. The versatility of this inverse design approach will be useful for a wide range of materials discovery and design problems.

## 4 Experimental Section

*Calculations of Composite Properties Using FEM*: The toughness of a composite is quantified as the amount of elastic energy that the composite can absorb prior to failure. The objective function and constraints in this composite design problem can be written as

(2) $$\min_{x}f(x)=-\sum_{i}E_{i}(x_{i})u_{i}^{T}(x)\;k_{0}u_{i}\;(x)$$

subject to

(3) $$E_{i}\;(x_{i})=E_{\mathrm{soft}}\;+x_{i}[E_{\mathrm{stiff}}-E_{\mathrm{soft}}]$$

(4) $$\frac{1}{n}\sum_{i}\;x_{i}=1-v^{*}$$

(5) $$E_{i}(x_{i})\;\varepsilon_{i,f}{\left(x_{i}\right)}^{2}=t^{*}$$

(6) $$0\leq x_{i}\leq 1,\;i=1,\ldots ,n$$

where *f* is the objective function to minimize, which is set to be the negative of the scaled toughness. ***x*** is the vector of *n* design variables, which are independent variables representing the type of base materials in each element. Here, the soft and stiff materials are denoted by 0 and 1, respectively. *E*_soft_ and *E*_stiff_ represent the moduli of the soft and stiff materials, respectively. To be able to calculate the gradients of the objective function with respect to the design variables, the design variables are set to be continuous with lower and upper bounds of 0 and 1 (side constraints), respectively. *E_i_* is the modulus of element *i* and ***u***_*i*_ is the displacement of element *i*. ***k***_0_ is the element stiffness matrix for an element with unit modulus. v* is the predetermined volume fraction. To ensure that the toughness of composites only depends on the geometrical configuration of base materials, the toughness of each element is set to be the same (independent of its modulus). t* is the predetermined toughness for each element. The toughness, strength, and stiffness of composites are calculated using FEM. A similar FEM analysis was adopted in our previous work.^8^ Linear elasticity is assumed in this composite design problem and the inclusion of nonlinear elasticity, plasticity, and crack propagation is left for future studies. More information about the composite models and FEM analysis is described in the Supporting Information.

*Inverse Design Using GIDNs*: The ML models are implemented and deployed using TensorFlow.^21^ GIDNs consist of two DNNs—the predictor and the designer. Both DNNs consist of six fully‐connected hidden layers with 256 neurons per layer. The activations of neurons are described as

(7) $$a_{j}^{\left(l+1\right)}=\sigma \left(\sum_{i}a_{i}^{\left(l\right)}w_{ij}^{\left(l,l+1\right)}+b_{j}^{\left(l+1\right)}\right)$$

where $a_{j}^{\left(l+1\right)}$ is the activation value of neuron *j* at layer *l*+1, which is the output of the weighted sum of the lower‐layer neurons passed through nonlinear activation function σ, $w_{ij}^{\left(l,l+1\right)}$ is the weight connecting neuron *i* at layer *l* and neuron *j* at layer *l*+1, and $b_{j}^{\left(l+1\right)}$ is the bias. The rectified linear unit (ReLU) is used as the activation function. The input of the predictor is a vector of 128 design variables representing the type of base materials in each element and the output is the predicted toughness. The predictor has around 360 000 learning parameters. To reduce overfitting and improve the generalization of the predictor, the dropout regularization with a rate of 0.5 is implemented in the training process. The Adam optimizer^22^ with a batch size of 10 000 is used to train the predictor for 1250 epochs. In the design process, the Adam optimizer is also used to update the design variable to minimize the objective function based on analytical gradients calculated using backpropagation. The objective function in the predictor to minimize during the training process is a lost function, which uses the mean squared error (MSE) to estimate the difference between the predicted toughness of training samples and the actual values calculated using FEM. On the other hand, the objective function in the designer to minimize during the design process is the negative of the toughness. The design variables in the designer are set to be continuous with lower and upper bounds of 0 and 1 and the mean value is set to match a predetermined volume fraction. The outputs of the designer are converted to binary values (0 and 1) based on the ranking of optimized design variables. The design process is terminated after 100 design loops. Compared to running with more design loops, it is found that this early stopping does not change the outputs much but significantly reduces the computational cost. The ML models are trained using the NVIDIA Tesla V100 and Titan V GPUs.

## Conflict of Interest

The authors declare no conflict of interest.

## Supporting information

Supporting Information
