# Supplementary material for: Generative Deep Neural Networks for Inverse Materials Design Using Backpropagation and Active Learning
Source: Adv Sci (Weinh). 2020 Jan 9;7(5):1902607. doi: 10.1002/advs.201902607 (PMC7055566; doi:10.1002/advs.201902607)
Supplement: Supplementary file 1 — Supporting Information [file ADVS-7-1902607-s001.pdf]

Copyright WILEY-VCH Verlag GmbH & Co. KGaA, 69469 Weinheim, Germany, 2020.

ADVANCED SCIENCE
Open Access

Supporting Information

for *Adv. Sci.,* DOI: 10.1002/advs.201902607

Generative Deep Neural Networks for Inverse Materials Design Using Backpropagation and Active Learning

*Chun-Teh Chen and Grace X. Gu**

SUPPORTING INFORMATION

Generative deep neural networks for inverse materials design using backpropagation and active learning

Chun-Teh Chen[1] and Grace X. Gu[2*]

[1] Department of Materials Science and Engineering, University of California, Berkeley, CA 94720, USA
[2] Department of Mechanical Engineering, University of California, Berkeley, CA 94720, USA
[*]Address correspondence to: ggu@berkeley.edu, +1.510.643.4996

SUPPORTING FIGURES

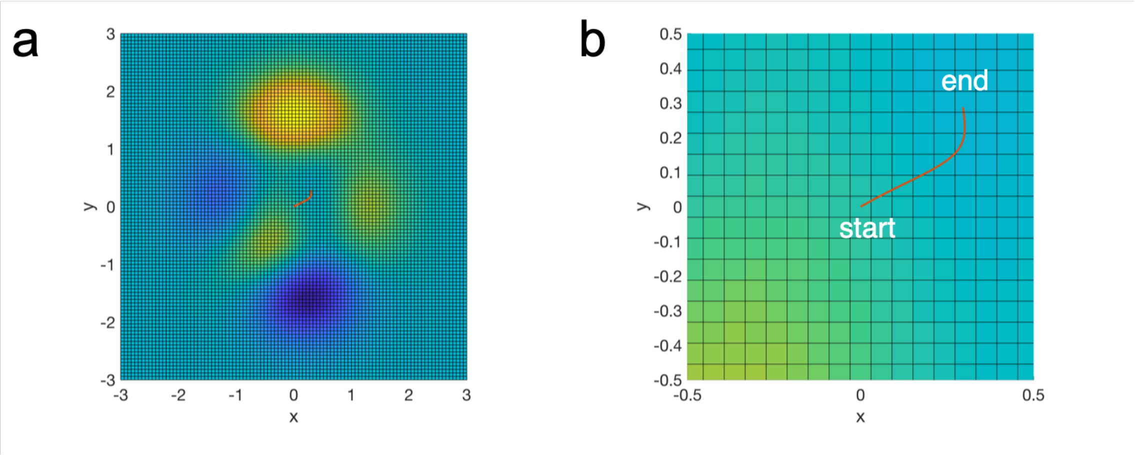

Fig. S1| 2D visualizations of the solution path using gradient descent. a, Visualization of the solution path (red line) when choosing the origin point ($x = 0$, $y = 0$) as the initial point. The optimization result converges to the nearest local minimum. **b,** Zoomed-in visualization of the solution path.

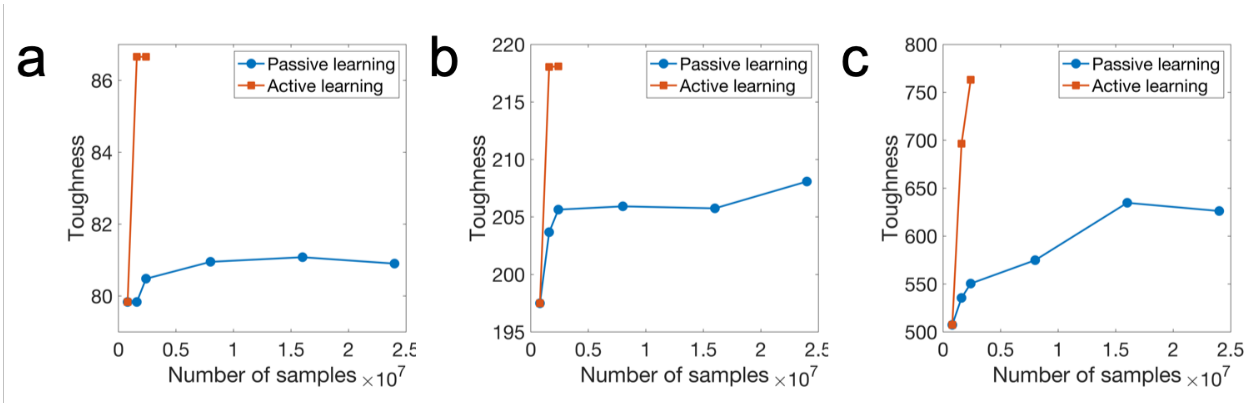

Fig. S2| Comparisons of passive and active learning strategies. a, Top performance comparison for the composite system with a volume fraction of 12.5%. **b,** Top performance comparison for the composite system with a volume fraction of 25%. **c,** Top performance comparison for the composite system with a volume fraction of 50%.

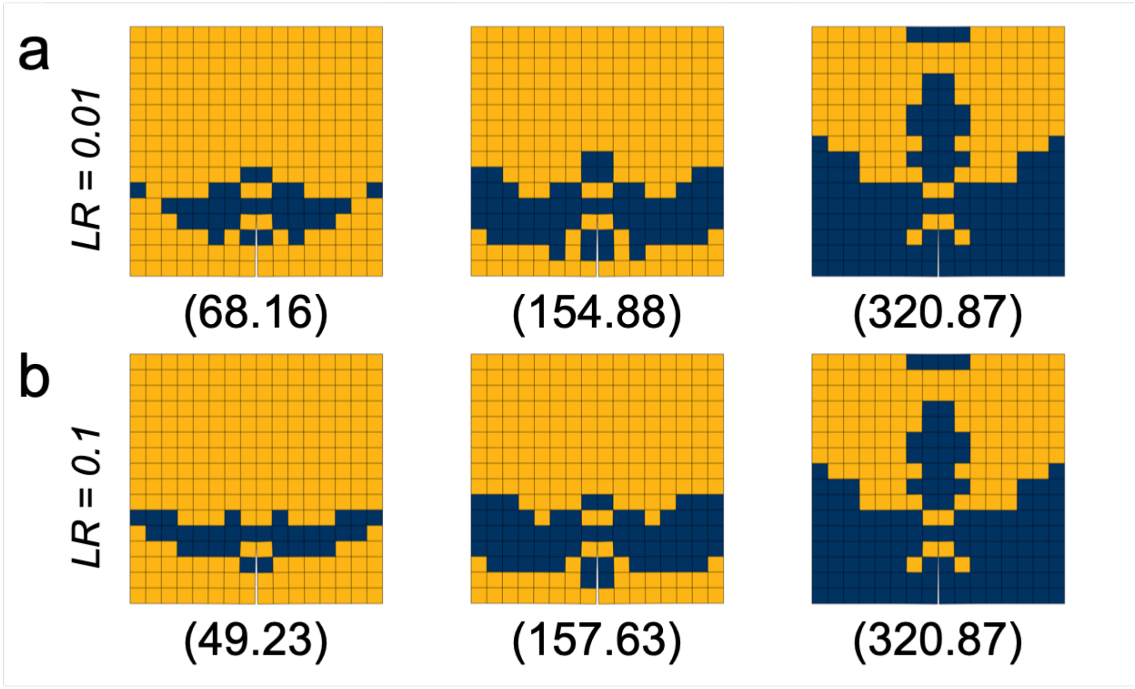

Fig. S3| Optimized designs generated by gradient-based topology optimization method. a, Optimized designs obtained using a learning rate of 0.01 for volume fractions of 12.5%, 25%, and 50%, respectively. **b,** Optimized designs obtained using a learning rate of 0.1 for volume fractions of 12.5%, 25%, and 50%, respectively.

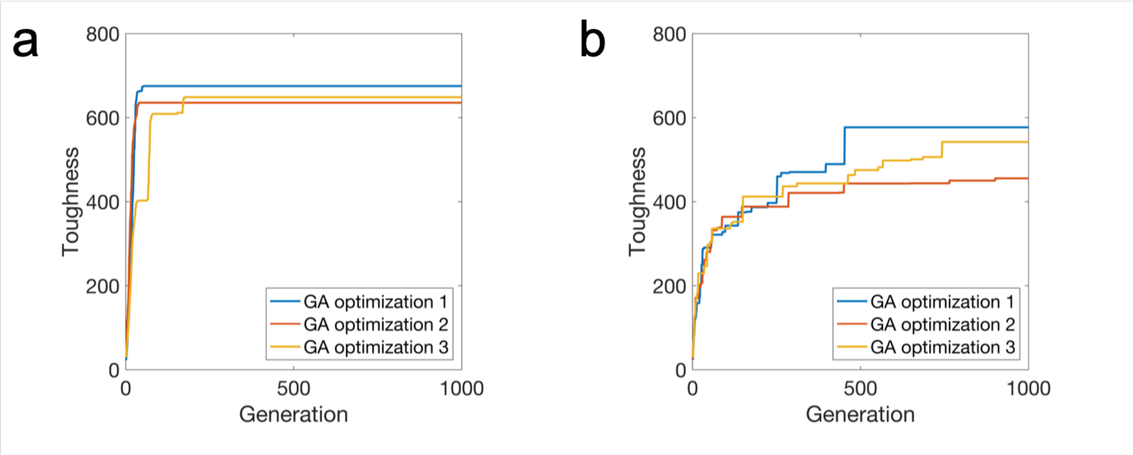

Fig. S4| Convergence of optimizations using genetic algorithm. a, Highest fitness score (toughness) in a population versus the generation number using a mutation rate of 0.01. **b,** Highest fitness score (toughness) in a population versus the generation number using a mutation rate of 0.1. Note that the optimization results will be different when running the optimization multiple times using a genetic algorithm. The same optimization is performed three times and the results are shown in different colors.

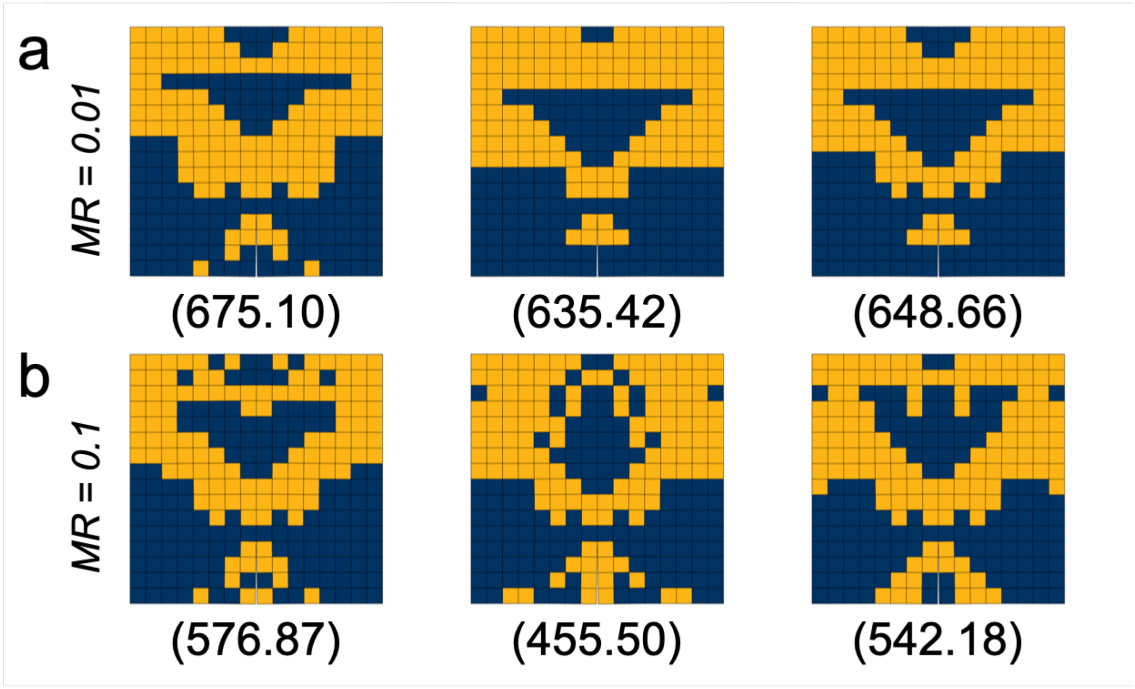

Fig. S5| Optimized designs obtained using genetic algorithm. a, Optimized designs obtained using a genetic algorithm with the mutation rate of 0.01. **b,** Optimized designs obtained using a genetic algorithm with the mutation rate of 0.1. Three optimized designs are generated by running the same optimization three times.

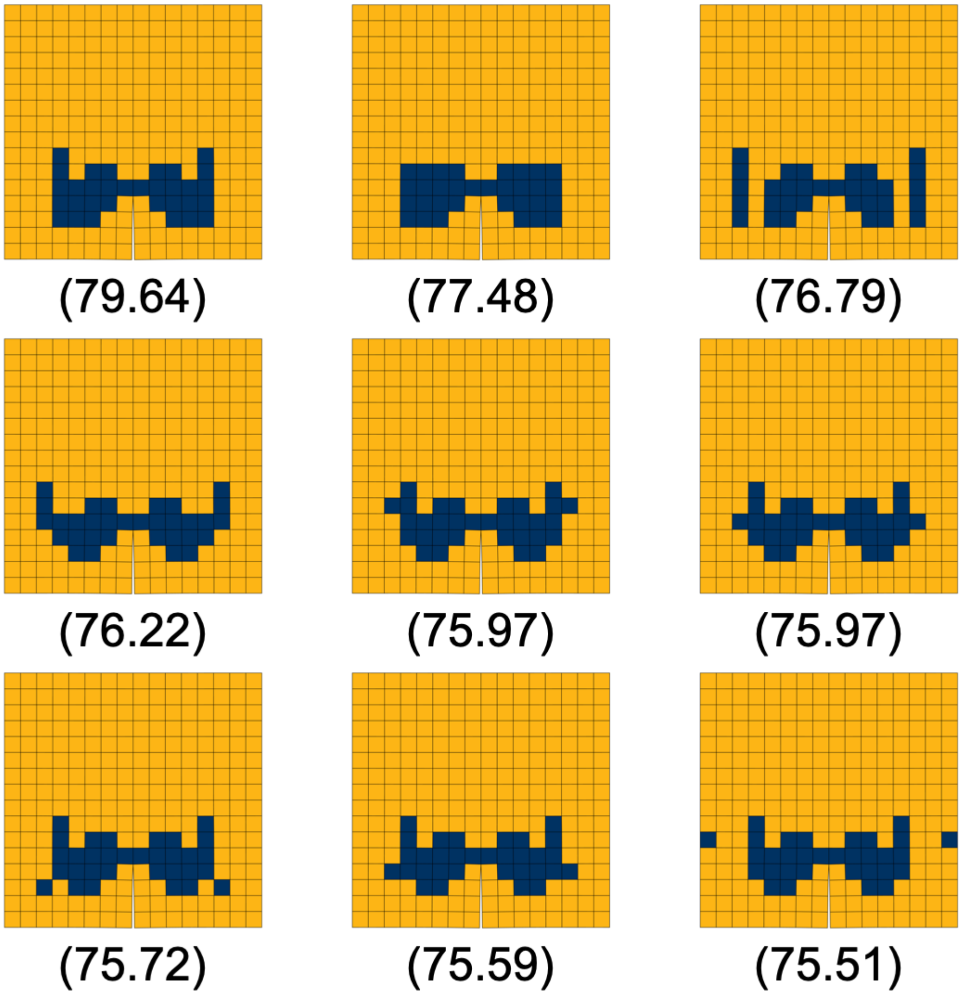

Fig. S6| Optimized designs obtained using logistic regression for volume fraction of 12.5%. These designs are the top 9 best design for high toughness in our previous work[1].

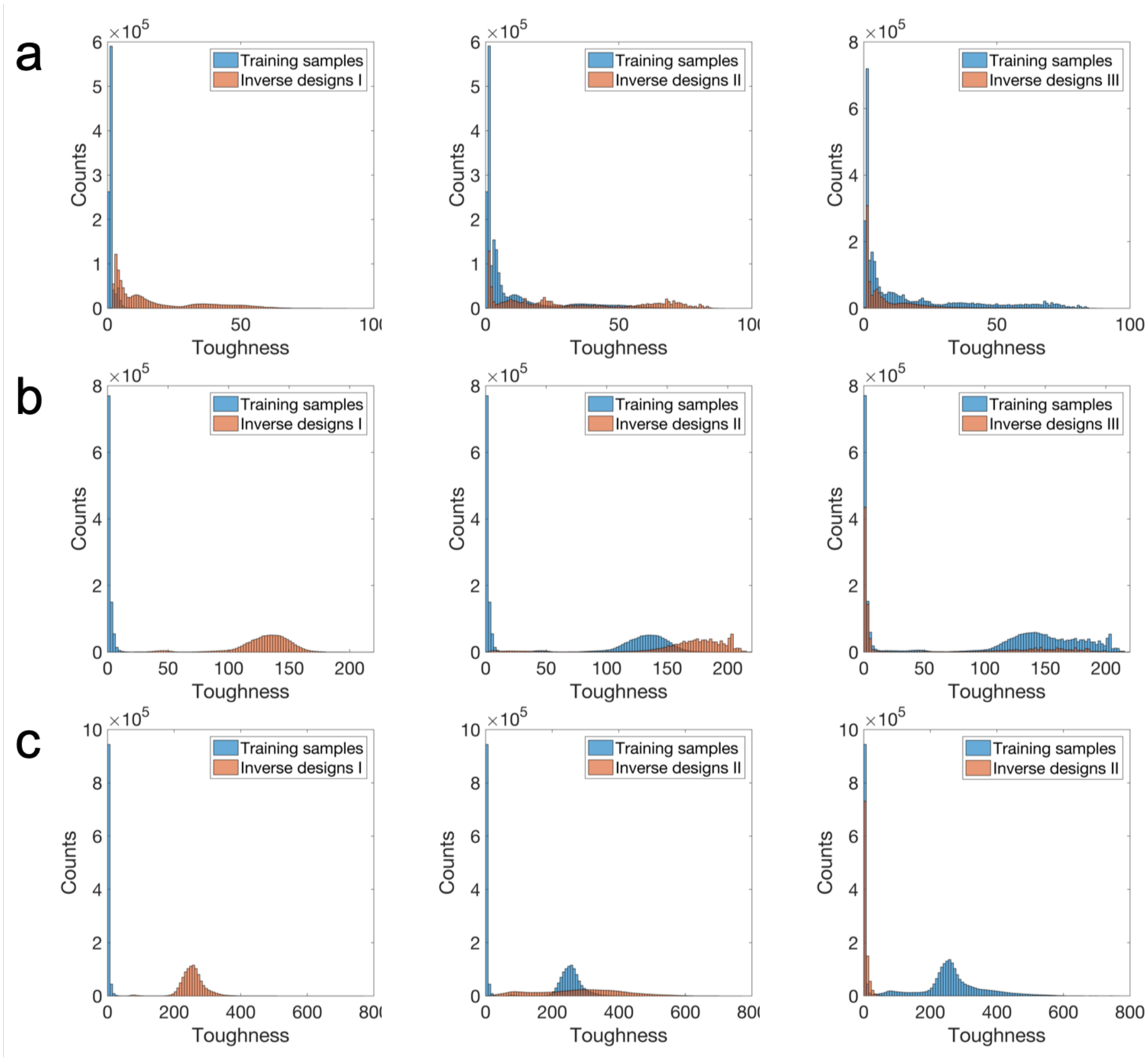

Fig. S7| Full histograms of training samples and inverse designs. a, Full histograms for the volume fraction of 12.5%. **b,** Full histograms for the volume fraction of 25%. **c,** Full histograms for the volume fraction of 50%. The subfigures from left to right are the histograms of the training samples and inverse designs for the first-iteration, second-iteration, and third-iteration.

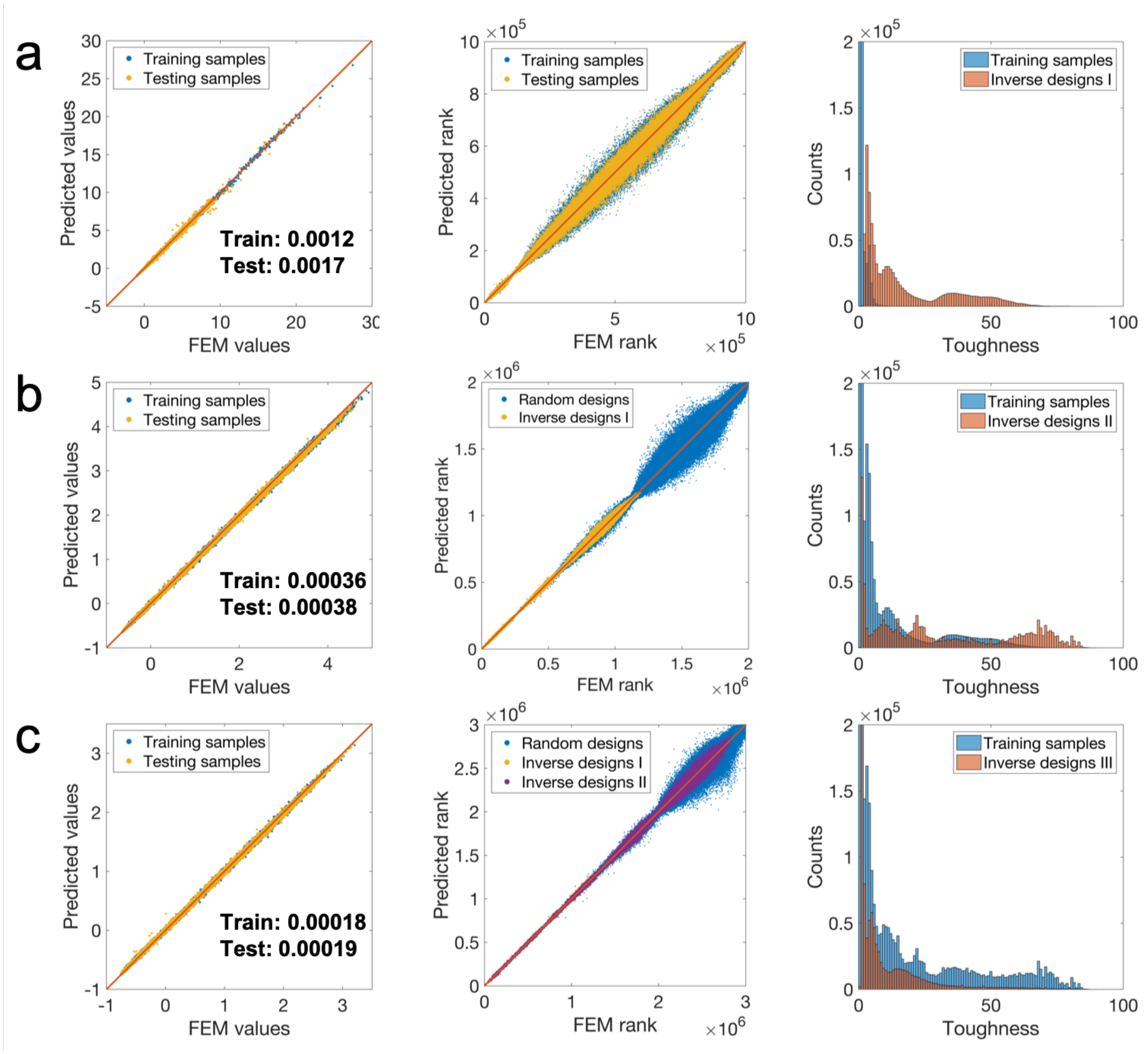

Fig. S8| Statistical analysis results for composite system with volume fraction of 12. 5 %. a, Statistical analysis results for the first-iteration. **b,** Statistical analysis results for the second-iteration. **c,** Statistical analysis results for the third-iteration. The subfigures from left to right are the comparison of the ML predicted values and FEM values, the comparison of the ML predicted ranking and FEM ranking, and the histogram of the training samples and inverse designs.

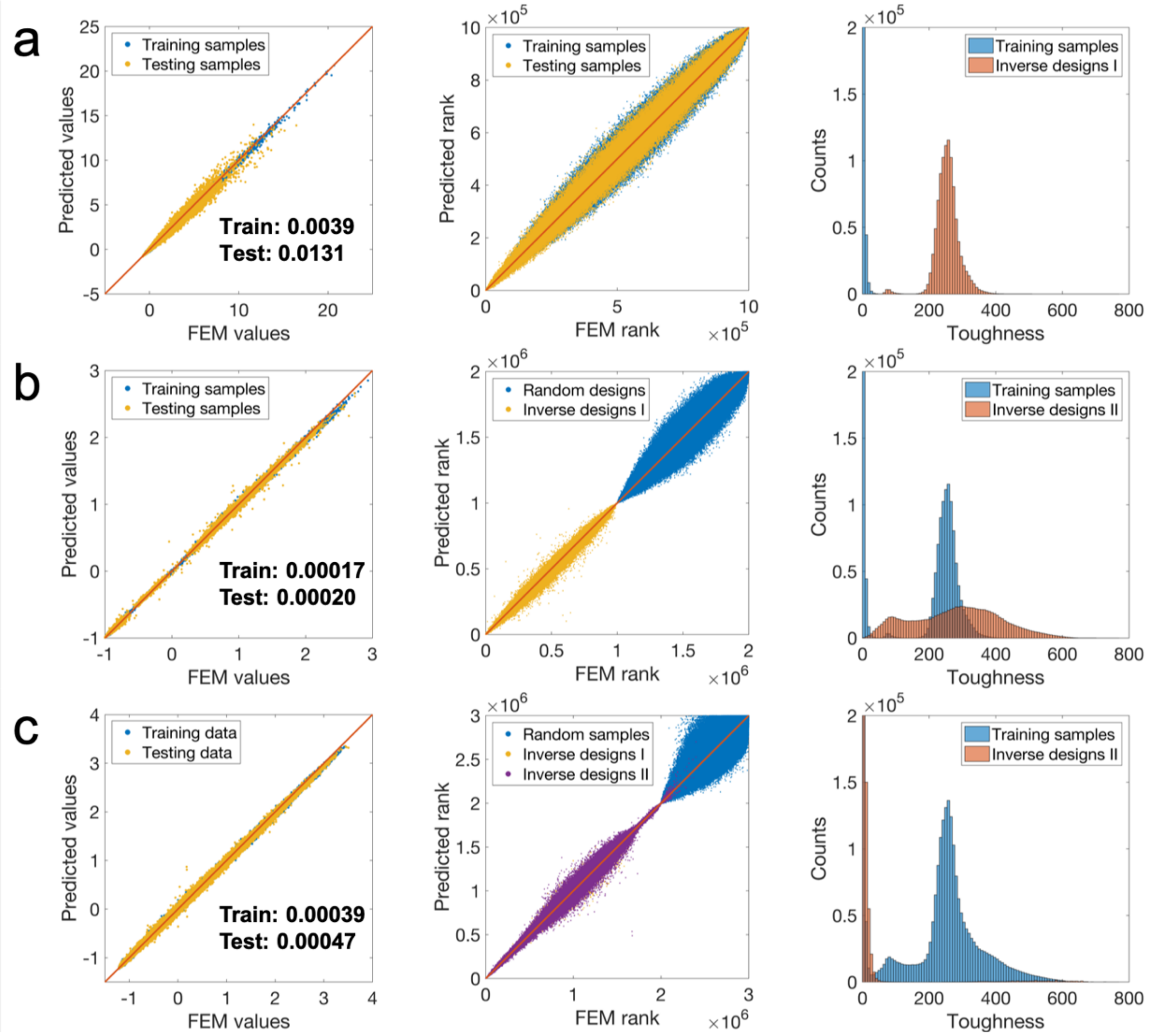

Fig. S9| Statistical analysis results for composite system with volume fraction of 50 %. a, Statistical analysis results for the first-iteration. **b,** Statistical analysis results for the second-iteration. **c,** Statistical analysis results for the third-iteration. The subfigures from left to right are the comparison of the ML predicted values and FEM values, the comparison of the ML predicted ranking and FEM ranking, and the histogram of the training samples and inverse designs.

Searching for peaks function's global minimum using GIDNs

The *predictor* and the *designer* consist of four fully-connected hidden layers with 64 neurons per layer. For the model, the rectified linear unit (ReLU) is used as the activation function. The input of the *predictor* is a vector of two variables (x and y) and the output is the predicted height (z). To reduce overfitting and improve the generalization of the *predictor*, the dropout regularization with a rate of 0.5 is implemented in the training process. 10,000 points from the peaks function are randomly generated, 8,000 of them are used as training samples to train the *predictor*, and the rest 2,000 are used as testing samples to evaluate its accuracy. The Adam optimizer with a batch size of 100 is used to train the *predictor* for 1,250 epochs. The comparison of the ML predicted height and ground truth is shown in **Fig. S10**. The training and testing errors are calculated as 0.00083 and 0.00085, respectivily. In the design process, 1,000 initial points are generated from a Gaussian distribution, in which the mean value is set to be zero and the standard deviation is set to be 1.5. Those initial points are then fed into the *designer* as inputs. The Adam optimizer is used to update the input variables to minimize the predicted height based on analytical gradients calculated using backpropagation.

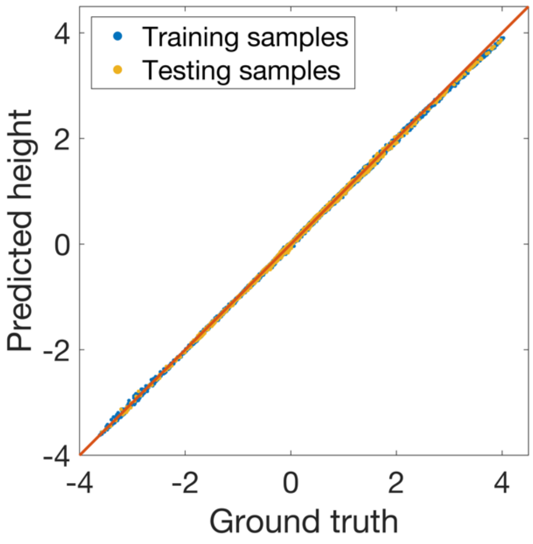

Fig. S10| Comparison of ML predicted height and ground truth.

Hyperparameter tuning for GIDNs

Hyperparameters related to neural network structures including the numbers of hidden layers and neurons are tuned to balance the prediction accuracy and computational cost. Four different numbers of hidden layers are considered: 2, 4, 6, and 8. Seven different numbers of neurons per layer are considered: 16, 32, 64, 128, 256, 512, and 1,024. Thus, a total of 28 neural network structures are created. Here, we use a composite system with a volume fraction of 25% to test the performance of those 28 ML models. 1,000,000 composite designs are randomly generated and their toughness values are calculated using FEM. 800,000 of them are used as training samples and the rest 200,000 are used as testing samples. A batch size of 10,000 is used to train those ML models for 1,250 epochs. After training, their prediction accuracy is shown in **Fig. S11**. The statistical analysis results are shown in **Fig. S12** to **S15**. As can be seen in the figures, the ML model with six hidden layers and 256 neurons per layer gives one of the lowest testing error (0.48) and outperforms other larger ML models. Therefore, we choose this set of hyperparameters to construct the GIDNs in the main paper.

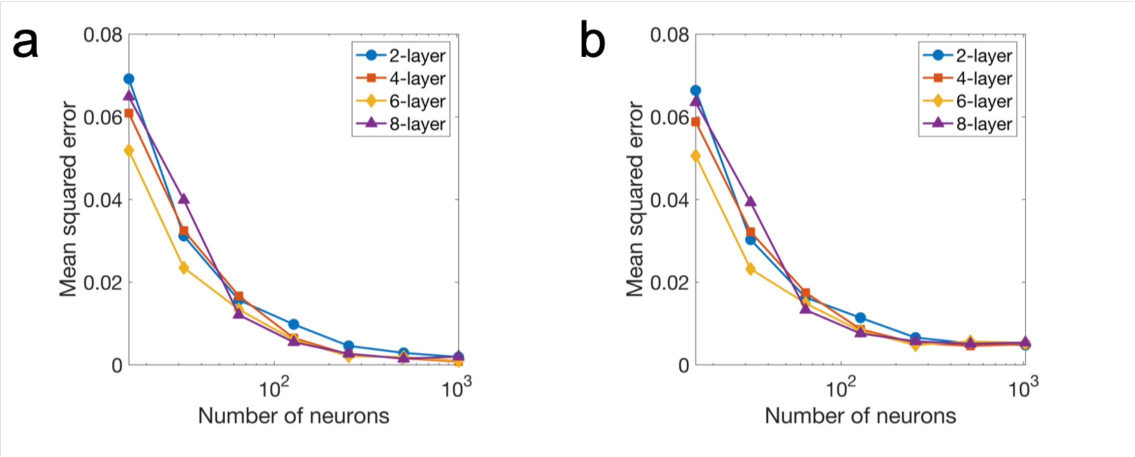

Fig. S11| Prediction accuracy of ML models with different neural network structures. a, Mean squared error for the training samples. **b,** Mean squared error for the testing samples.

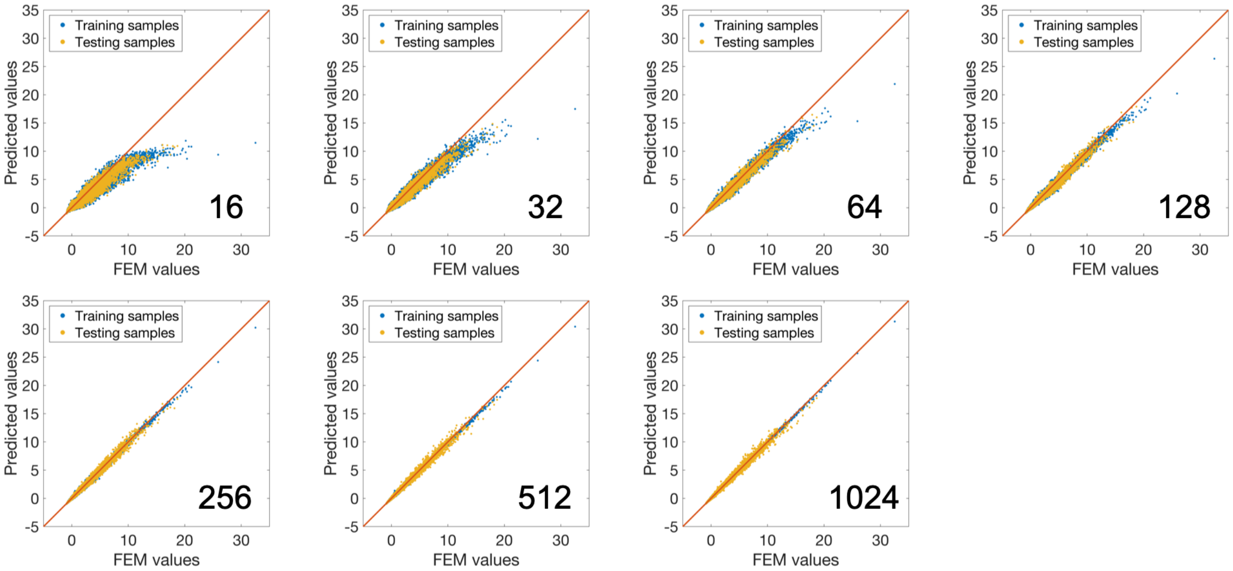

Fig. S12| Comparisons of ML predicted values and FEM values for neural network structures with two hidden layers. The number in each subfigure represents the number of neurons used per layer.

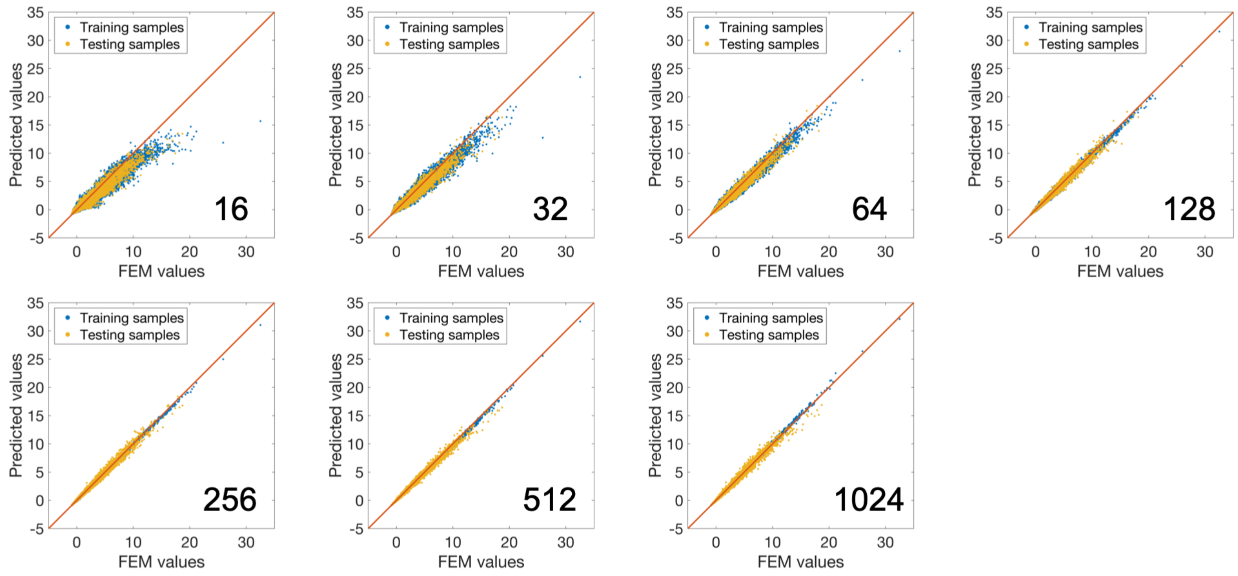

Fig. S13| Comparisons of ML predicted values and FEM values for neural network structures with four hidden layers. The number in each subfigure represents the number of neurons used per layer.

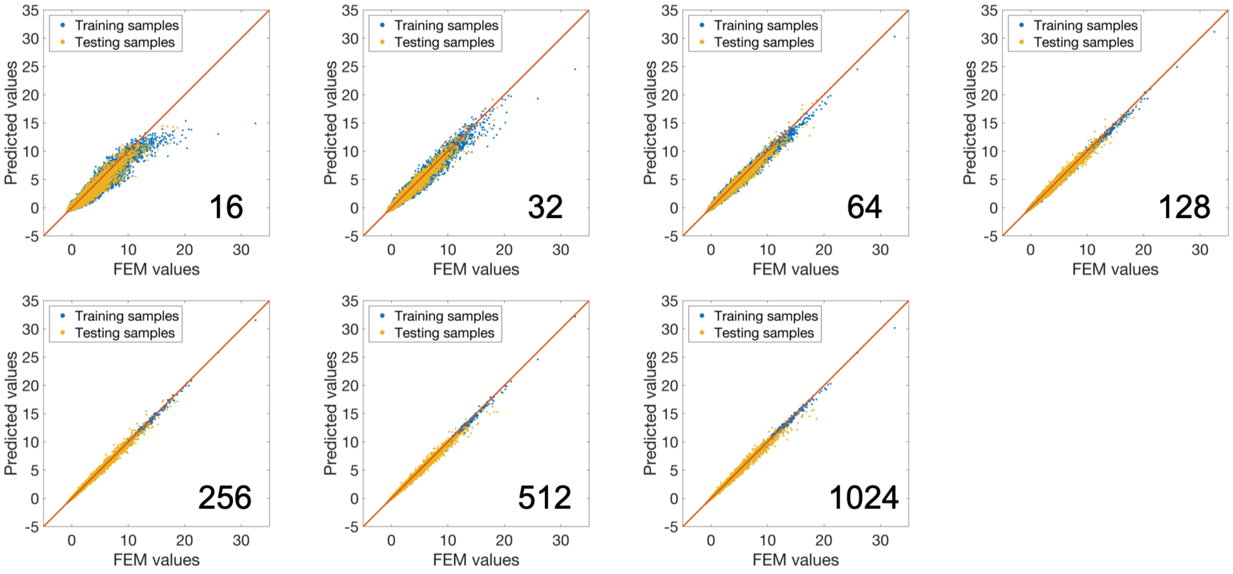

Fig. S14| Comparisons of ML predicted values and FEM values for neural network structures with six hidden layers. The number in each subfigure represents the number of neurons used per layer.

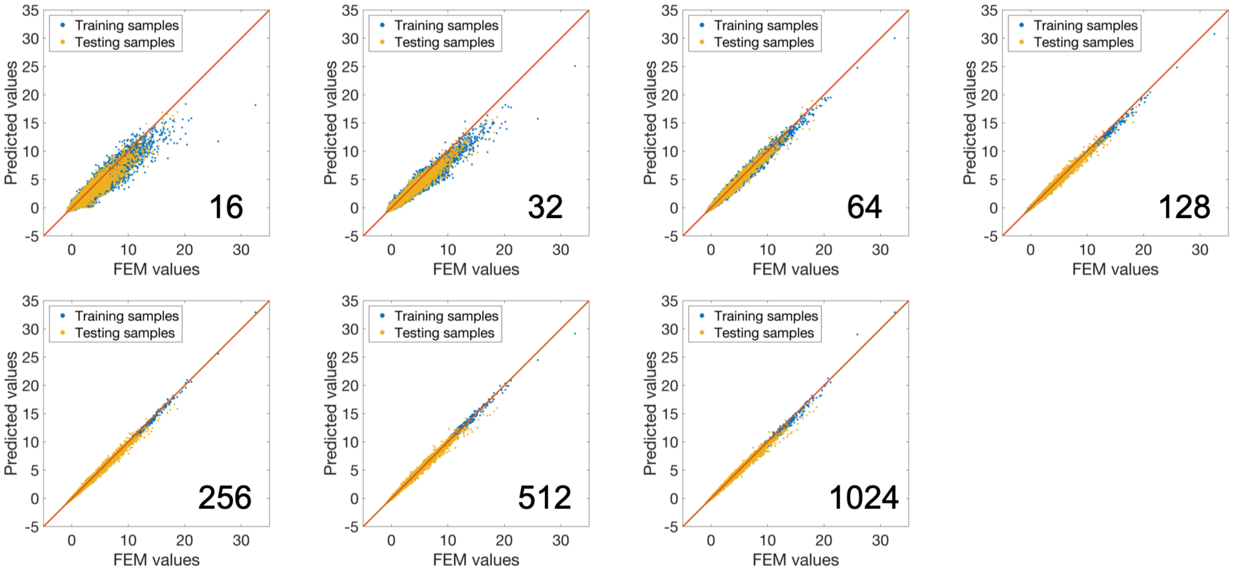

Fig. S15| Comparisons of ML predicted values and FEM values for neural network structures with eight hidden layers. The number in each subfigure represents the number of neurons used per layer.

FEM analysis for composites using four-node elements

The composite design domain is discretized by square elements as shown in **Fig. S16**. Four-node elements are implemented with an assumption that the failure of elements occurs in the linear elastic regime. The composites are considered to be made up of perfectly brittle linear elastic materials, in which materials do not exhibit yielding (plastic deformation) before failure. The toughness of such material can be quantified as the amount of elastic energy per unit volume that it can absorb prior to failure, which can be written as:

$$T = \int_0^{\varepsilon_f} \sigma d\varepsilon = \frac{E\varepsilon_f^2}{2} = \frac{\sigma_f^2}{2E} \quad \text{(Eq. S1)}$$

where *T* is the toughness, *E* is the modulus, ε_f is the failure strain, and σ_f is the failure stress (material strength). An edge crack, which is 25% of the specimen width in the y-direction, is created by the insertion of double nodes. Additionally, displacement boundary conditions are applied along the *x*-direction to simulate *Mode I* fracture. Geometrical symmetry is assumed in the system since the edge crack is located at the centerline of the specimen and the loading condition is symmetric. The modulus of the stiff material (E_{stiff}) is set to 1 GPa and the failure strain (ε_f) is set to 10%. Since the modulus ratio of base materials is set to 10, the modulus of the soft material (E_{soft}) is set to 0.1 GPa. To ensure the stiff and soft materials have the same toughness, the failure strain of the soft material is set to $\sqrt{10}\%$. The Poisson ratio for both stiff and soft materials is set to $1/3$.

After applying displacement boundary conditions, the strain in the loading direction (ε_{xx}) at the crack tip is used to calculate the toughness and strength of a composite. Once the strain reaches the failure strain (ε_f) of crack-tip elements, the composite is considered to have failed and its toughness (the area underneath the stress-strain curve) and strength (maximum stress) can be determined. The resistance of composites during crack propagation is not considered here; instead, due to computational limitations, we consider the resistance of composites to initiate crack propagation.

The stiffness matrix of the four-node elements is:

$$\begin{Bmatrix} F_{x1} \\ F_{y1} \\ F_{x2} \\ F_{y2} \\ F_{x3} \\ F_{y3} \\ F_{x4} \\ F_{y4} \end{Bmatrix} = \begin{bmatrix} C_1 & & & & & & & \\ C_2 & C_3 & & & & & & \\ C_4 & -C_5 & C_1 & & & sym. & & \\ C_5 & C_6 & -C_2 & C_3 & & & & \\ -\frac{C_1}{2} & -C_2 & C_7 & -C_5 & C_1 & & & \\ -C_2 & -\frac{C_3}{2} & C_5 & C_8 & C_2 & C_3 & & \\ C_7 & C_5 & -\frac{C_1}{2} & C_2 & C_4 & -C_5 & C_1 & \\ -C_5 & C_8 & C_2 & -\frac{C_3}{2} & C_5 & C_6 & -C_2 & C_3 \end{bmatrix} \begin{Bmatrix} u_1 \\ v_1 \\ u_2 \\ v_2 \\ u_3 \\ v_3 \\ u_4 \\ v_4 \end{Bmatrix}$$

$$C_1 = \left(\frac{b}{3a} + \frac{1-\nu}{6}\frac{a}{b}\right)\frac{Et}{1-\nu^2}$$
$$C_2 = \left(\frac{\nu}{4} + \frac{1-\nu}{8}\right)\frac{Et}{1-\nu^2}$$
$$C_3 = \left(\frac{a}{3b} + \frac{1-\nu}{6}\frac{b}{a}\right)\frac{Et}{1-\nu^2}$$
$$C_4 = \left(-\frac{b}{3a} + \frac{1-\nu}{12}\frac{a}{b}\right)\frac{Et}{1-\nu^2}$$
$$C_5 = \left(\frac{\nu}{4} - \frac{1-\nu}{8}\right)\frac{Et}{1-\nu^2}$$
$$C_6 = \left(\frac{a}{6b} - \frac{1-\nu}{6}\frac{b}{a}\right)\frac{Et}{1-\nu^2}$$
$$C_7 = \left(\frac{b}{6a} - \frac{1-\nu}{6}\frac{a}{b}\right)\frac{Et}{1-\nu^2}$$
$$C_8 = \left(-\frac{a}{3b} + \frac{1-\nu}{12}\frac{b}{a}\right)\frac{Et}{1-\nu^2}$$

where a is the element length in the x-direction, b is the element length in the y-direction, ν is the element Poisson ratio, E is the element modulus, and t is the element thickness.

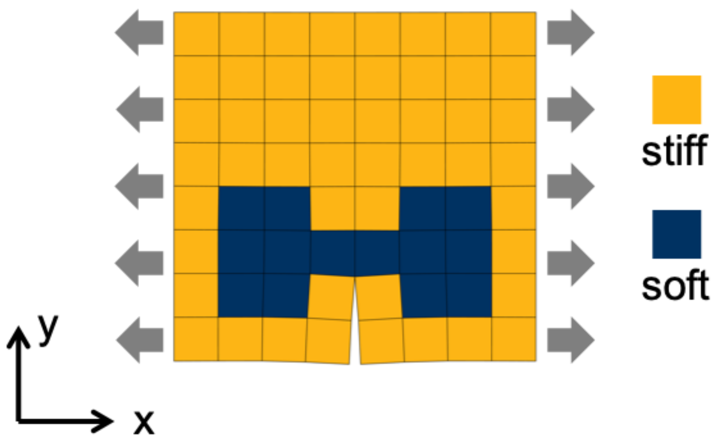

Fig. S16| Composite FEM model made up of stiff and soft elements. Stiff elements are shown in yellow and soft elements are shown in blue. An edge crack, which is 25% of the specimen width in the y-direction is created by the insertion of double nodes. Displacement boundary conditions are applied along the x-direction to simulate *Mode I* fracture.

Gradient-based topology optimization

To apply a gradient-based topology optimization method to the composite design problem, the design variables are set to be continuous with lower and upper bounds set to 0 (as the soft material) and 1 (as the stiff material), respectively. To ensure that the toughness of composites only depends on the geometrical configuration of base materials, the toughness of each element is set to be the same (independent of its modulus). A schematic diagram of the stress-strain curves for elements having different moduli is shown in **Fig. S17**.

The objective function and constraints adopted in topology optimization are the same as those shown in **Eq. 2**. The objective function is evaluated using FEM. The optimization steps are as follows:

1. **Initialize design variables:** The design variables are initialized based on the predetermined volume fraction constraint. For example, all the initial values are set to be 0.875 for the volume fraction of 12.5%.

2. **Calculate the gradient vector:** The numerical gradient vector of the objective function with respect to the design variables is calculated using FDM with the central difference approximation:

$$\nabla f(\mathbf{x}) = g(\mathbf{x}) = \begin{Bmatrix} \dfrac{\partial f}{\partial x_1} \\ \vdots \\ \dfrac{\partial f}{\partial x_{128}} \end{Bmatrix} \cong \begin{Bmatrix} \dfrac{f(x_1 + h, \dots, x_{128}) - f(x_1 - h, \dots, x_{128})}{2h} \\ \vdots \\ \dfrac{f(x_1, \dots, x_{128} + h) - f(x_1, \dots, x_{128} - h)}{2h} \end{Bmatrix}$$

   where h represents a small change in design variables. Here, h is set to be 10^{-5}.

3. **Normalize the gradient vector:** The gradient vector is scaled to a range of 0 and 1 and then normalized to a mean of zero.

4. **Update the design variables:** The design variables for the next optimization step $(i + 1)$ is updated toward the negative of the normalized gradient vector:

$$\mathbf{x}_{i+1} = \mathbf{x}_i - \lambda \hat{g}(\mathbf{x}_i)$$

   where λ is the step size, also known as the learning rate. Here, two λ values are considered: 10^{-2} and 10^{-1}.

5. **Repeat until convergence:** Repeat steps 2, 3, and 4 until the optimization result is converged. Here, the number of iterations is set to be 10^5.

6. **Convert to binary values:** The optimized result is converted to binary values (0 and 1) based on the ranking of optimized design variables.

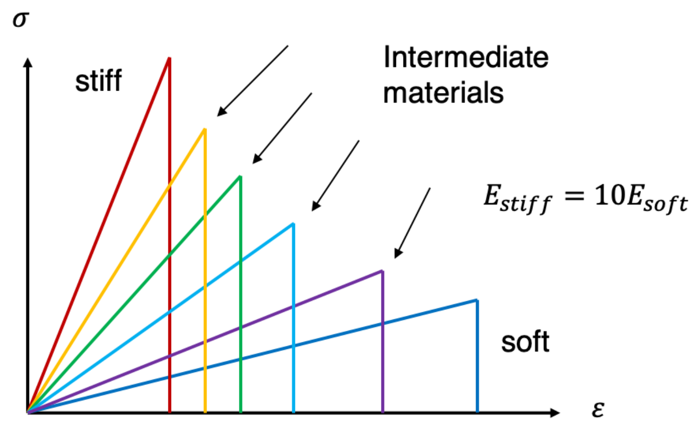

Fig. S17| Stress-strain curves of elements with different moduli. Each element can be the stiff material or soft material, or intermediate material, which has the modulus in between the stiff material and soft material. However, the toughness of each element, which is defined as the area underneath the stress-strain curve, is set to be the same.

Gradient-free genetic algorithms

A gradient-free genetic algorithm is applied to the composite design problem. The fitness function is set to be the toughness, which is evaluated using FEM. The optimization steps are as following:

1. **Initialize population:** The individuals in the initial population are generated randomly, in which the genes (design variables) are either 0 or 1. Here, the population size is set to be 10^3.

2. **Calculate the fitness scores:** The fitness scores of the individuals are calculated using FEM. The fitness score determines the probability of an individual to be selected for reproduction.

3. **Select best individuals for mating:** The individuals with the highest fitness scores are selected as parents to pass their genes to the next generation. Here, the number of parents is set to be 10^2.

4. **Operate crossover:** A center crossover point is selected and the tails of the two parents are swapped to produce new offspring.

5. **Operate mutation:** A low mutation probability is applied to the genes of new offspring, which allows each of the genes to switch its value from 0 to 1 (switch from the soft material to the stiff material) and vice versa. Here, two mutation probabilities are considered: 10^{-2} and 10^{-1}.

6. **Repeat until convergence:** Repeat steps 2, 3, 4, and 5 until the population is converged. Here, the number of iterations is set to be 10^5. Additionally, we terminate the algorithm after 10^3 generations.

Performance of inverse designs for 8 by 8 composite system

To estimate the amount of training data required for GIDNs to identify global minima of highly complex design problems. The 8 by 8 composite system with a volume fraction of 25% is adopted for the investigation as the optimal designs of this system were already identified using a brute-force search in our previous work.[2] This composite system has a total of 10,518,300 possible combinations. The neural network structures of the GIDNs for this composite system consist of six fully-connected hidden layers with 64 per layer. Five different training groups are used to train the *predictor*. The numbers of training samples in each training group are 100, 1,000, 10,000, 100,000, 1,000,000, respectively. The maximum and mean toughness values of the training groups are shown in **Table S1** and **Fig. S18a**. Note that the toughness values are normalized by the toughness of a composite made up of all stiff (or soft) material. It can be seen that the maximum toughness value increases as the number of training samples increases. However, the mean toughness values are close to each other. Note that none of the training groups includes the optimal design (Composite-A in **Fig. 3**), which has a toughness value of 69.4. After training for 1,250 epochs, 1,000,000 initial designs generated from a Gaussian distribution are fed into the *designer* as inputs. The performance of the optimized designs generated by the *designer* when the *predictor* is trained with different training groups is shown in **Table S2** and **Fig. S18b**. In general, the performance of the optimized designs increases with the number of training samples used to train the *predictor*. Furthermore, the *designer* is able to identify the optimal design when the *predictor* is trained with 1,000 or more samples. When the *predictor* is trained with only 100 samples, the *designer* has failed to identify the optimal design. However, the second-best design with a toughness value of 65.13 (Composite-B in **Fig. 3**) is identified by the *designer*. The results show that the proposed inverse design approach only requires a small amount of training data in order to the optimal design of the 8 by 8 composite system.

Table S1. Performance of training samples in different training groups. The numbers of training samples in each training group are 100, 1,000, 10,000, 100,000, 1,000,000, respectively. The toughness values are normalized by the toughness of a composite made up of all stiff (or soft) material.

	100 samples	1,000 samples	10,000 samples	100,000 samples	1,000,000 samples
Max value	11.75	29.00	31.58	62.68	62.68
Mean value	1.91	1.92	1.89	1.91	1.91

Table S2. Performance of optimized designs generated by *designer* when *predictor* is trained with different training groups. The values of top 1% and top 10% represent the mean toughness values of the top 1% and top 10% optimized designs, respectively.

	100 samples	1,000 samples	10,000 samples	100,000 samples	1,000,000 samples
Max value	65.13	69.42	69.42	69.42	69.42
Top 1%	61.61	62.68	62.73	64.85	65.18
Top 10%	43.51	61.85	62.24	61.64	63.47
Mean value	18.56	34.45	33.37	31.40	35.33

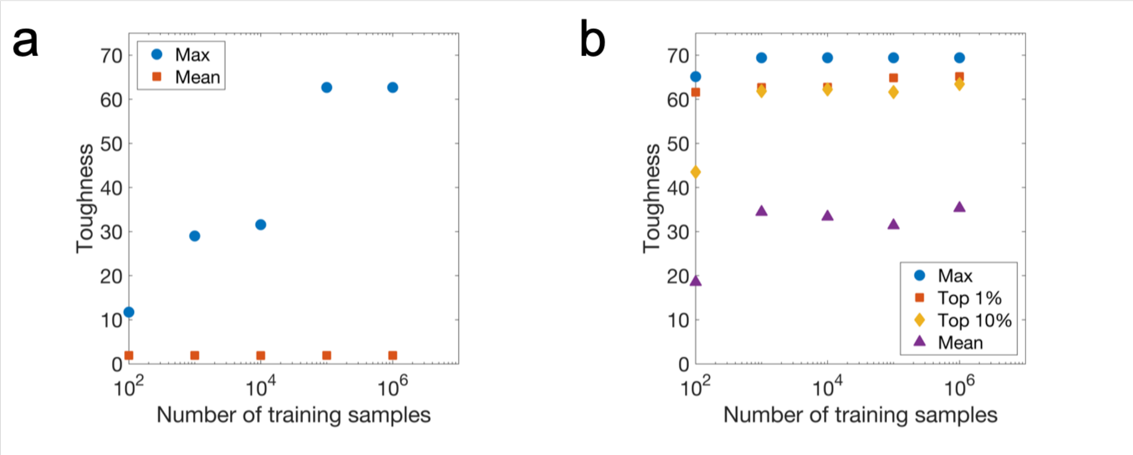

Fig. S18| Performance of optimized designs generated by using different numbers of training samples. a, Maximum and mean toughness values of different training groups. **b** Performance of the optimized designs generated by using different training groups.

REFERENCES

[1] G. X. Gu, C.-T. Chen, M. J. Buehler, Extreme Mechanics Letters 2018, 18, 19.
[2] C.-T. Chen, G. X. Gu, Advanced Theory and Simulations 2019, 2, 1900056.
